# Application of the Chinese Version of the Pittsburgh Sleep Quality Index in People Living With HIV: Preliminary Reliability and Validity

**DOI:** 10.3389/fpsyt.2021.676022

**Published:** 2021-07-06

**Authors:** Dong-Qin Yan, Yun-Xiang Huang, Xi Chen, Min Wang, Jie Li, Dan Luo

**Affiliations:** ^1^Department of Social Medicine and Health Management, Xiangya School of Public Health, Central South University, Changsha, China; ^2^Chinese Evidence-Based Medicine Center, West China Hospital, Sichuan University, Chengdu, China; ^3^Hunan Provincial Center for Disease Prevention and Control, Changsha, China; ^4^Human Immunodeficiency Virus/Acquired Immunodeficiency Syndrome Research Institute, The First Hospital of Changsha, Changsha, China; ^5^Furong District Center for Disease Prevention and Control, Changsha, China

**Keywords:** pittsburgh sleep quality index, reliability, validity, HIV, sleep disturbances, China

## Abstract

**Background:** The Pittsburgh Sleep Quality Index (PSQI) has been a widely used instrument measuring sleep quality among people living with HIV (PLWH) in China while its psychometric properties have yet to be examined in this population. We aimed to assess the reliability and validity of the Chinese version of PSQI in PLWH and identify factors associated with sleep quality.

**Methods:** This study was based on a longitudinal study of newly diagnosed PLWH, among whom the PSQI was used to measure sleep quality 5 years after HIV diagnosis (*n* = 386). To evaluate internal consistency, Cronbach's alpha and corrected item-total correlation were calculated. To assess construct validity, Pearson's correlation coefficients were calculated between PSQI scores and depression, anxiety, stress, and health-related quality of life (HRQoL). Known group validity was evaluated by comparing PSQI scores between participants with probable depression and those without. Binary logistic regression was conducted to identify factors associated with sleep disturbances.

**Results:** The internal consistency Cronbach's alpha for the Chinese version of PSQI in PLWH was 0.713. Construct validity was established by significant relationships between PSQI and depression, anxiety, stress, and HRQoL. The PSQI scores in participants with probable depression were significantly higher than those without, indicating good known-group validity. Sleep disturbances were associated with less income, higher CD4 counts, antiretroviral treatment (ART) initiation, exercise, depression, and higher stress levels.

**Conclusions:** The Chinese version of PSQI is feasible for use among Chinese PLWH. Over a third of PLWH reported sleep disturbances. More attention should be given to individuals with less income and on ART. Intervention aimed at improving mental health or facilitating exercise may improve sleep quality.

## Introduction

Receiving diagnosis of HIV infection is considered as an extremely stressful experience for most individuals, accompanied with considerable stressors such as stigma, disclosure, emotional distress, medication side effects, and declines in physical function (1, 2), which are known to be strong predictors of sleep disturbances among people living with HIV (PLWH) (3, 4). PLWH are, therefore, more likely to be vulnerable to sleep problems (5). A meta-analysis estimating the prevalence of self-reported sleep disturbances among PLWH found that approximately more than half of PLWH report sleep disturbances after diagnosis (5), while PLWH complaining of poor sleep quality have been shown to be less likely to adhere to recommended treatment and more likely to suffer multiple mental disorders (6, 7), which may negatively impact the immune and virologic responses (8), leading to treatment failure (9) and, ultimately, influencing the quality of life in this population (10).

Given the significantly negative consequence of the poor sleep quality among PLWH, providing a reliable estimate of the prevalence of sleep disturbances has been increasingly important. Sleep quality is evaluated either by self-reported or interviewer-rated scales or objective measures (such as polysomnography and actigraphy) (11). Empirical evidence showed that self-reported measures are user-friendly, reliable, and sensitive to change in sleep pattern and quality (12). Of the different measures on sleep quality, the Pittsburgh Sleep Quality Index (PSQI) is the most widely used (5). The Chinese version of the PQSI was translated in 1996 by Liu et al. and have been subsequently examined in different populations including the civil servants, college students, and rural elderly (13), all indicating the Chinese version PSQI is a reliable and valid instrument for evaluating sleep quality (14–16).

Although previous studies have explored various psychometric properties of the Chinese-version PSQI across different clinical and non-clinical groups, its application in PLWH has yet to be examined. This study aimed to (1) examine the reliability and validity of the Chinese version of the PQSI in PLWH, and (2) assess the prevalence of poor sleep quality using PQSI and identify the factors associated with poor sleep quality among PLWH.

## Methods

### Participants

Participants were HIV-infected patients enrolled since 2013 in a longitudinal study designed to evaluate mental health challenges, among people with newly diagnosed HIV infection. Relevant description on the study design is available in elsewhere (17). Briefly, individuals with newly diagnosed HIV were consecutively recruited from the Changsha Center for Disease Control and Prevention, Hunan Province, China. Individuals were eligible if they were (1) receiving HIV diagnosis for less than 1 month (newly diagnosed with HIV), (2) more than 18 years of age, and (3) having lived in Changsha city for more than 6 months. This study was approved by the Ethics Committee of Xiangya School of Public Health Central South University (ZYGW-2018-055). Written informed consent was obtained from each participant before participation.

A total of 855 people newly diagnosed with HIV met the inclusion criteria, among which 557 participants completed the baseline survey between March 1, 2013 and September 30, 2014. After 1 year, 410 participants continued to participate in the follow-up survey. Among the 557 individuals who completed the baseline survey, 386 agreed to participate in the 5-year follow-up survey which was conducted between August 1, 2018 and March 29, 2019. This study was based on data from 5-year follow-up survey in which the sleep quality was added as a new variable of interest.

### Measures

#### Socio-Demographic Information

Demographic information included gender, age (18–29, 30–39, or ≥40), marriage status (single, married, or divorced/widowed), education (senior or below, college or above), employment (employed or unemployed), monthly income (≤ 4,000 Yuan or >4,000 Yuan), and exercise behavior (yes or no).

#### Sleep Quality

Sleep quality was assessed by the Chinese version of PSQI, which was translated and validated by Liu et al. in 1996 (13). The original scale was designed by Buysse et al. which was used to measure the sleep quality and disturbances over the past month (12). It includes 18 items consisting of seven components: subjective quality of sleep, sleep latency, sleep duration, habitual sleep efficiency, sleep disturbance, use of sleep medication, and daytime dysfunction. Each item is scored from 0 (not during the past month) to 3 (three or more times a week), with total score ranging from 0 to 21. A higher score suggests poorer sleep quality. A cut-off score of 5 has been recommended to screen for sleep disturbance by Buysse et al. (12).

#### Clinical Information

Clinical information including CD4 counts and antiretroviral treatment (ART) status were obtained from the Chinese HIV/AIDS Comprehensive Response Information Management System. In addition, participants were asked in questionnaire whether they had any other disease (except for HIV infection).

#### Depression

Depression was assessed by the 9-item Patient Health Questionnaire Depression Scale (PHQ-9) (18). Participants responded on a 4-point Likert-type scale ranging from 0 (not at all) to 3 (nearly every day). The total score of scare ranges from 0 to 27, with a score ≥10 being considered a cut-off screening for significant depressive symptoms (19). The Chinese version of the PHQ-9 shows good reliability and validity with a Cronbach's α coefficient of 0.86 (20).

#### Anxiety

Anxiety was measured by the 7-item Generalized Anxiety Disorder questionnaire (GAD-7) (21). Each item was rated on a 4-point Likert scale ranging from 0 (not at all) to 3 (nearly every day). The total score of scale ranges from 0 to 21. A score of 10 points or higher was usually used as cut-off to identify significant anxiety symptoms (22). The Chinese version of GAD-7 shows good reliability and validity with a Cronbach's α coefficient of 0.88 (23).

#### Stress

The Chinese version of HIV/AIDS Stress Scale (CSS-HIV) was used to assess HIV-related stress (23). It was first developed by Pakenham et al. (24), and Niu et al. later translated it into Chinese version. This scale consists of three subscales: social stress, instrumental stress, and emotional stress. Participants were asked how much stress they had endured in the past month on a 5-point Likert-type scale. A higher score indicates higher levels of stress. In this study, the median score of the CSS-HIV (*P*_50_ = 13) was used as the cut-off to divide low and high stress. The CSS-HIV has good validity and reliability, with an overall Cronbach's α coefficient of 0.906 (23).

#### Health-Related Quality of Life

The health-related quality of life was measured using the Medical Outcomes Study HIV Survey (MOS-HIV) (25). The MOS-HIV includes 35 items with 11 dimensions containing general health, physical function, role function, cognitive function, pain, mental health, health distress, energy/fatigue, social function, overall quality of life, and health transition. Based on standard scoring procedures, a physical health summary score and a mental health summary score could be calculated. The Chinese version of the MOS-HIV has shown good validity and reliability among PLWH (26).

### Statistical Analysis

The continuous variables were described as median with interquartile ranges (IQRs) and categorical variables were described as numbers with percentages. To evaluate the internal consistency, we calculated Cronbach's alpha for overall scale and corrected item-total correlation, with Cronbach's α ≥0.70 and corrected correlations ≥0.30 indicating adequate internal consistency (27). To evaluate construct validity, Pearson's correlation coefficients were calculated between PSQI scores and other theoretically related constructs, i.e., depression, anxiety, stress, and HRQoL. Known group validity was evaluated by comparing each component scores of PSQI between participants with probable depression (PHQ-9 ≥ 10) and without depression (PHQ-9 < 17) using independent sample *t* test. We hypothesized that depression is negatively associated with poor sleep quality. To identify the factors associated with sleep disturbances (defined as PSQI > 5), univariate logistical regression was conducted, with experiencing sleep disturbance or not as dependent variable, and sex, age, marital status, education, employment, monthly income, CD4 count, comorbidities, ART status, exercise, HIV-related stress, depressive, and anxiety symptoms as independent variables. Variables that were statistically associated with sleep disturbances with a *p* ≤ 0.2 in the univariate logistic regression were further selected into the multiple logistic regression model. Odds ratios (OR) and 95% CIs were presented. All analyses were conducted using SPSS 22.0 (SPSS Inc., Chicago, IL, USA) with two-tailed *p* < 0.05 considered statistically significant.

## Results

### Sample Characteristics

Of the 386 participants who completed the 5-year follow-up survey after diagnosis, 353 (91.5%) were male, with a median age of 34 (IQR: 30–43). The majority were single, employed, and had a monthly income of more than 4,000 Yuan. Nearly two-thirds of participants self-reported as homosexual or bisexual. The median CD4 counts 5 years after diagnosis were 512 cells/mm^3^ (IQR: 368–656). Over one-third of individuals reported the presence of other disease except for HIV infection (Table 1).

**Table 1:** Sample characteristics.

**Variables**	***n***	**%**
Sex
Male	353	91.5
Female	33	8.5
Age, median (IQR)	34	(30–43)
18–29	94	24.4
30–39	171	44.3
≥40	121	31.3
Marital status
Single	189	49.0
Married	138	35.8
Divorced/widowed	59	15.2
Education
Senior or lower	215	55.7
College or higher	171	44.3
Employment
Employed	318	82.4
Unemployed	68	17.6
Monthly income (Yuan), median (IQR)	5,000	(3,000–6,500)
≤ 4,000	179	46.4
>4,000	207	53.6
Exercise
No	212	54.9
Yes	174	45.1
Sexual orientation
Heterosexuality	142	36.8
Homosexuality	155	40.2
Bisexuality	89	23.1
CD4 count (cells/mm^3^), median (IQR)	512	(368–656)
≤ 350	84	21.8
>350	302	78.2
Comorbidities
No	265	68.7
Yes	121	31.3
ART		
No	36	9.3
Yes	350	90.7
Depressive symptoms
No	320	82.9
Yes	66	17.1
Anxiety symptoms
No	347	89.9
Yes	39	10.1

*ART, antiretroviral treatment*.

### Reliability

The corrected item-total correlation ranged from 0.131 for the use of sleep medication component to 0.702 for the subjective sleep quality component. The overall Cronbach's α was 0.719 and increased to 0.734 after excluding the use of sleep medication component (Table 2).

**Table 2:** PSQI internal consistency data.

**PSQI component**	**Corrected item-total correlation**
Subjective sleep quality	0.702
Sleep latency	0.497
Sleep duration	0.462
Habitual sleep efficiency	0.380
Sleep disturbance	0.414
Sleep medication use	0.131
Daytime dysfunction	0.469
Cronbach's α	0.719

*PSQI, Pittsburgh Sleep Quality Index*.

### Validity

#### Construct Validity

Correlations between the PSQI scores and relevant variables are presented in Table 3. Theoretical related constructs such as depression, anxiety, stress, and HRQoL were all significantly correlated with the total score of the PSQI (*r* ≥ |0.360|). Each component scores of the PSQI were significantly correlated with the depression, anxiety, and mental health summary scores of HRQoL (|*r*| = 0.110–0.526, *p* < 0.05). In addition, all components of the PSQI were correlated with the scores of stress the physical health summary scores of HRQoL (|*r*| = 0.107–0.369, *p* < 0.05), except for the component of sleeping medication use (|*r*| = 0.042–0.065, *p* > 0.05).

**Table 3:** Correlations between sleep measures and relevant variables.

**PSQI component**	**Depression**	**Anxiety**	**Stress**	**HRQoL**	**HRQoL**
				**(PHS)**	**(MHS)**
Subjective sleep quality	0.426^**^	0.354^**^	0.317^**^	−0.301^**^	−0.439^**^
Sleep latency	0.319^**^	0.306^**^	0.255^**^	−0.236^**^	−0.332^**^
Sleep duration	0.266^**^	0.222^**^	0.177^**^	−0.211^**^	−0.329^**^
Habitual sleep efficiency	0.143^**^	0.130^*^	0.107^*^	−0.116^*^	−0.209^**^
Sleep disturbance	0.267^**^	0.219^**^	0.264^**^	−0.260^**^	−0.302^**^
Use of sleep medication	0.157^*^	0.179^**^	0.042	−0.065	−0.110^*^
Daytime dysfunction	0.456^**^	0.336^**^	0.316^**^	−0.334^**^	−0.455^**^
Total score of the PSQI	0.486^**^	0.409^**^	0.360^**^	−0.369^**^	−0.526^**^

* *p < 0.05;*

** *p < 0.01.*

*MHS, mental health summary score; PHS, physical health summary score; PSQI, Pittsburgh Sleep Quality Index*.

#### Known Group Validity

As hypothesized, the total score of the PSQI in individuals with probable depression (PHQ-9 ≥ 10, *n* = 66; mean = 6.42, *SD* = 3.90) was significantly higher than that in normal individuals with no depression (PHQ-9 <10, *n* = 320; mean = 3.50, *SD* = 3.50) with *p* < 0.001 (Table 4). All of the PSQI components, except for two components, sleep efficiency and use of sleep medication, were significantly correlated with the depression (*p* < 0.001).

**Table 4:** PSQI scores according to the PHQ-9.

**PSQI component**	**No depression**		**Probable depression**		***t***	***p***
	**Mean**	**SD**	**Mean**	**SD**		
Subjective sleep quality	0.55	0.63	1.09	0.76	−6.077	<0.001
Sleep latency	0.82	0.99	1.33	1.19	−3.721	<0.001
Sleep duration	0.40	0.86	0.88	1.17	−3.857	<0.001
Habitual sleep efficiency	0.30	0.66	0.42	0.81	−1.365	0.173
Sleep disturbance	0.67	0.58	1.00	0.61	−4.163	<0.001
Use of sleep medication	0.03	0.22	0.08	0.40	−1.431	0.153
Daytime dysfunction	0.73	0.94	1.62	1.17	−6.647	<0.001
Total score of the PSQI	3.50	3.05	6.42	3.90	−6.741	<0.001

*PHQ-9, 9-item Patient Health Questionnaire Depression Scale; PSQI, Pittsburgh Sleep Quality Index*.

### Sleep Quality Among PLWH

#### Prevalence and Severity of Symptoms

The global PSQI score for the 386 participants ranged from 0 to 18, with a median of 3 (IQR: 1–6). The overall prevalence rate of poor sleep quality was 37.0%, using a cut-off point of 5, as suggested by Buysse et al. (12).

#### Factors Associated With Sleep Quality in PLWH

In the multivariate regression analysis, we found participants with less income (*p* = 0.002), CD4 >350 cells/mm^3^ (*p* = 0.024), ART initiation (*p* = 0.047), exercise behavior (*p* = 0.008), depressive symptoms (*p* = 0.006), and higher stress levels (*p* = 0.032) were more likely to experience sleep disturbances (Table 5).

**Table 5:** Factors associated with sleep quality in PLWH.

**Variables**	**Univariate**		**Multivariate**	
	**OR (95% CI)**	***p***	**OR (95% CI)**	***p***
Sex
Male	Ref			
Female	1.12 (0.54–2.32)	0.770		
Age
18–29	Ref			
30–39	0.84 (0.50–1.41)	0.503		
≥40	0.81 (0.47–1.42)	0.463		
Marital status
Married	Ref		Ref	
Single	1.24 (0.78–1.97)	0.355	1.60 (0.93–2.73)	0.088
Divorced/widowed	1.74 (0.94–3.25)	0.081	1.93 (0.97–3.83)	0.060
Education
Senior or lower	Ref			
College or higher	0.79 (0.52–1.19)	0.257		
Employment
Unemployed	Ref		Ref	
Employment	0.65 (0.38–1.10)	0.110	1.08 (0.56–2.07)	0.824
Monthly income (Yuan)
≤ 4,000	Ref		Ref	
>4,000	0.47 (0.31–0.72)	<0.001	0.44 (0.26–0.74)	0.002
CD4 count (cells/mm^3^)
≤ 350	Ref		Ref	
>350	1.63 (0.96–2.75)	0.071	2.00 (1.10–3.66)	0.024
Comorbidities
No	Ref		Ref	
Yes	1.37 (0.88–2.13)	0.161	1.10 (0.66–1.82)	0.722
ART
No	Ref		Ref	
Yes	2.23 (0.94–5.29)	0.070	2.63 (1.01–6.82)	0.047
Exercise
No	Ref		Ref	
Yes	0.45 (0.29–0.69)	<0.001	0.53 (0.33–0.85)	0.008
Depressive symptoms
No	Ref		Ref	
Yes	4.47 (2.54–7.85)	<0.001	2.83 (1.34–5.98)	0.006
Anxiety symptoms				
No	Ref		Ref	
Yes	3.47 (1.74–6.92)	<0.001	1.43 (0.59–3.43)	0.427
HIV-related stress				
Low	Ref		Ref	
High	3.01 (1.96–4.63)	<0.001	1.75 (1.05–2.92)	0.032

*ART, antiretroviral treatment; PLWH, people living with HIV*.

## Discussion

Although the PSQI has been frequently used in the studies of PLWH in China, the reliability and validity of the Chinese version in this population has yet to be examined. This is the first study to examine the psychometric efficiency of the PSQI among Chinese PLWH. We found that the Chinese version of the PSQI has adequate reliability and validity in PLWH. Over a third of PLWH reported sleep disturbances (defined as PSQI >5) in this study. Individuals who had a lower income, higher CD4 counts, ART initiation, exercise behavior, presence of depressive symptoms, and higher stress levels were more likely to experience sleep disturbances.

The reliability of PSQI in this study was supported by high internal consistency (Cronbach's α = 0.719), which was close to the result from studies by Buysse et al. (12). The component on sleep medication use had an item-total correlation <0.3 in this study, indicating poor correlations for this component in the PSQI framework. After deleting the component on sleep medication use, the Cronbach's α coefficient of PSQI increased from 0.719 to 0.734. Such a result is consistent with several published studies (28, 29). Of note, removing the medication use component was based on psychometrical methods, whereas the best possible psychometrics may not always be the highest consideration.

The validity of PSQI was supported by good construct validity and known-group validity. Significant correlations between PSQI and theoretical related constructs such as depression, anxiety, stress, and HRQoL were found in this study. In addition, the total score of PSQI in individuals with probable depression (PHQ-9 ≥ 10) was found to be significantly higher than that of individuals with no depression (PHQ-9 <10), compatible with our hypothesis that individuals with depression are more likely to experience poor sleep quality. The reliability and validity analyses of PSQI in this study suggest that the Chinese version of PSQI is a suitable and acceptable instrument for use in assessing sleep quality among Chinese PLWH.

Poor sleep quality was observed in 37% of participants in this study, which is much higher than that of the general population survey in China where the rate of sleep disturbances was found to be about 10% (30). This result, however, was lower than the rate of 43.1% reported by another study conducted among 4,103 HIV-infected individuals at 20 AIDS clinics across China (31). The different lengths of time since diagnosis may partially explain the discrepancy between the two studies. The median duration of diagnosis was 2.25 years in that study and 5 years in our sample, while the length of time since diagnosis has been associated with sleep disturbances, with shorter duration from diagnosis being associated with poor sleep quality (31). Nevertheless, the prevalence of sleep disturbances remains high even 5 years after diagnosis. Routinely assessing sleep quality over the course of the HIV infection should be taken into consideration. In accordance, identifying factors associated with sleep disturbances among PLWH is critical to inform strategies to improve sleep quality among this population.

In this study, PLWH on ART were more likely to report sleep disturbances. It is well-known that the morbidity and mortality rates among PLWH have declined dramatically with the scale-up of ART (32). However, side effects associated with ART have also been frequently reported by patients on ART, with sleep disturbances as a common side effect of treatment (33). A previous study investigating factors influencing adherence to ART mentioned that 56.4% of patients regarded insomnia as an adverse effect of ART, which further contribute to the non-adherence and discontinuation of treatment (34). Some antiretroviral medications (e.g., efavirenz) have been linked to adverse sleep effects, especially at higher plasma levels (35). Regularly monitoring adverse reactions to ART should be an important consideration in the management of HIV.

Our study found that individuals with higher CD4 counts (>350 cells/mm^3^) have a higher rate of sleep disturbances when compared with those with lower CD4 counts (≤ 350 cells/mm^3^). Conversely, findings from most HIV studies suggested higher CD4 counts were associated with better sleep quality (36, 37). It seems a more likely scenario as it would be expected that as patients decrease their viral load and improve their CD4 counts, their overall health would improve, together with sleep. However, a study in South Africa where 79% of the participants were women also reported a similar relationship between higher CD4 counts and poor sleep quality, arguing this may be related to an underlying immune activation (38). In addition, a cross-sectional study conducted in France found that patients with CD4 count <500 cells/mm^3^ were more likely to be long sleepers and less likely to experience insomnia. Insomnia and impaired sleep quality seem to be highly prevalent in well-controlled PLWH (39). Further investigation is needed to explore the relationship between CD4 counts and sleep quality.

5Poor immune function, serious symptoms, and antiretroviral side effects in the 1990s were broadly considered as the contributors of sleep disturbances in PLWH (40, 41), while even in the context of improved antiretroviral therapy and optimally controlled viral replication, PLWH still struggle with sleep disturbances, indicating that sleep disturbances among PLWH may be caused by additional factors related to psychosocial status other than HIV disease. Individuals would suffer from a myriad of stressors related to HIV after being diagnosed, such as disclosure concerns and infection-related stigma (2), which may make individuals with HIV infection be burdened further by depression and sleep disturbances (42, 43). Consistent with previous studies that depression is a major factor influencing sleep quality among PLWH (44, 45), we found that individuals with depression were more likely to experience sleep disturbances than those without. Considering the possible bidirectional association between sleep and depression (46), alleviating depressive symptoms among PLWH may improve sleep quality and vice versa.

In our study, less income was significantly associated with increased risk of sleep disturbances, and this variable is known to be an important factor associated with sleep whatever the medical condition (47). In addition, the beneficial effect of exercise on sleep has been commonly demonstrated among the general population or patients with other disease such as cancer survivors, and people with rheumatoid arthritis and mental illness (48–51), while less attention has been paid to PLWH. In this study, we found PLWH with exercise behavior were less likely to report sleep disturbances. Further research should investigate which type of exercise and exercise intensity is more effective for the sleep quality of PLWH.

Several limitations of this study should be acknowledged. First, consecutive sampling was used in this study to recruit participants, which may limit the generalizability of the findings. Second, other factors that may influence sleep quality were not included in this study, e.g., pain, alcohol assumption, smoking, and body mass index (BMI). These variables should be considered in future studies. Another limitation is that the sleep quality among PLWH in this study was based on one-time point assessment. Longitudinal studies tracking sleep disturbances among PLWH throughout the course of the disease could be valuable to characterize the impact of HIV infection on sleep.

## Conclusions

The findings from this study supported the feasibility of the PSQI for use among Chinese PLWH. Over a third of PLWH reported sleep disturbances in this study, and participants with less income, higher CD4 counts, ART, exercise behavior, depressive symptoms, and higher stress levels were more likely to experience sleep disturbances. More attention should be given to the screening and treatment for sleep disturbances experienced by PLWH.

## Data Availability Statement

The raw data supporting the conclusions of this article will be made available by the authors, without undue reservation.

## Ethics Statement

This study was approved by the Ethics Committee of Xiangya School of Public Health Central South University (ZYGW-2018-055). Written informed consent was obtained from each participant before participation.

## Author Contributions

D-QY: investigation, data curation, formal analysis, visualization, writing—original draft, writing—review and editing. Y-XH: investigation, data curation, formal analysis, visualization, writing—original draft, writing—review and editing, supervision. DL: conceptualization, methodology, project administration, funding acquisition, writing—review and editing, supervision. XC: methodology, writing—review, and editing. MW and JL: methodology, writing—review and editing. All authors contributed to the article and approved the submitted version.

## Conflict of Interest

The authors declare that the research was conducted in the absence of any commercial or financial relationships that could be construed as a potential conflict of interest.
